# Crystal structure of 2-amino-4-phenyl-4*H*-benzo[*h*]chromene-3-carbo­nitrile

**DOI:** 10.1107/S2056989015011536

**Published:** 2015-06-27

**Authors:** Shaaban K. Mohamed, Peter N. Horton, Mehmet Akkurt, Sabry H. H. Younes, Mustafa R. Albayati

**Affiliations:** a Chemistry and Environmental Division, Manchester Metropolitan University, Manchester M1 5GD, England; b Chemistry Department, Faculty of Science, Minia University, 61519 El-Minia, Egypt; c School of Chemistry, University of Southampton, Highfield, Southampton SO17 1BJ, England; d Department of Physics, Faculty of Sciences, Erciyes University, 38039 Kayseri, Turkey; e Chemistry Department, Faculty of Science, Sohag University, 82524 Sohag, Egypt; f Kirkuk University, College of Science, Department of Chemistry, Kirkuk, Iraq

**Keywords:** crystal structure, amino­chromene, fused chromene, hydrogen bonding, C—H⋯π inter­actions

## Abstract

In the title compound, C_20_H_14_N_2_O, the plane of the phenyl ring is almost normal to that of the naphthalene ring system, forming a dihedral angle of 83.15 (8)°. The 4*H*-pyran ring fused with the naphthalene ring system has a flattened boat conformation. In the crystal, mol­ecules are linked by pairs of N—H⋯N hydrogen bonds, forming inversion dimers with an *R*_2_^2^(12) ring motif. The dimers are connected by C—H⋯π inter­actions, forming supra­molecular chains along [010].

## Related literature   

For synthesis of chromene-containing compounds, see: Elagamey *et al.* (1988 ▸); El-Maghraby (2014 ▸). For industrial applications of amino­chromenes, see: Ellis (1977 ▸); Hafez *et al.* (1987 ▸). For various biological activities of fused chromenes, see: Hiramoto *et al.* (1997 ▸); Bianchi & Tava (1987 ▸); Eiden & Denk (1991 ▸); Smith *et al.* (1998 ▸); Taylor *et al.* (1998 ▸). For the crystal structure of the isomer of the title compound, 3-amino-1-phenyl-1*H*-benzo[*f*]chromene-2-carbo­nitrile, see: Akkurt *et al.* (2013 ▸).
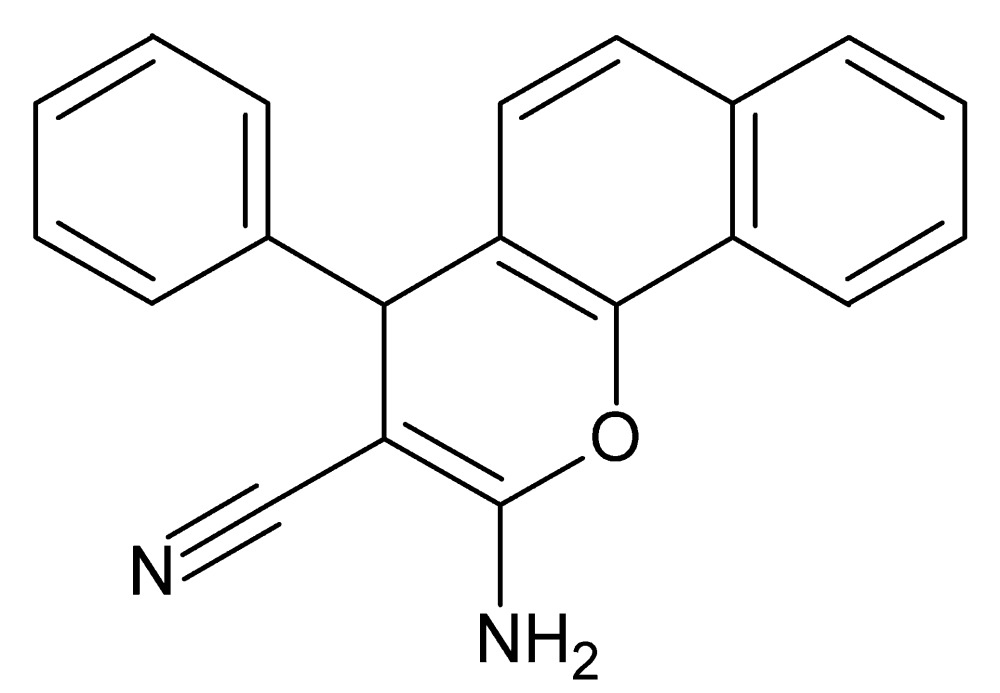


## Experimental   

### Crystal data  

C_20_H_14_N_2_O
*M* *_r_* = 298.33
Monoclinic, 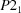

*a* = 9.1662 (1) Å
*b* = 5.7246 (1) Å
*c* = 13.9177 (2) Å
β = 90.153 (1)°
*V* = 730.30 (2) Å^3^
*Z* = 2
Cu *K*α radiation
μ = 0.67 mm^−1^
*T* = 100 K
0.28 × 0.13 × 0.10 mm

### Data collection  

Rigaku AFC11 diffractometer
Absorption correction: multi-scan (*CrystalClear-SM Expert*; Rigaku, 2012 ▸) *T*_min_ = 0.883, *T*_max_ = 1.000
5778 measured reflections
2201 independent reflections
2184 reflections with *I* > 2σ(*I*)
*R*_int_ = 0.029

### Refinement  

*R*[*F* ^2^ > 2σ(*F* ^2^)] = 0.031
*wR*(*F* ^2^) = 0.093
*S* = 1.09
2201 reflections
216 parameters
1 restraint
H atoms treated by a mixture of independent and constrained refinement
Δρ_max_ = 0.14 e Å^−3^
Δρ_min_ = −0.15 e Å^−3^
Absolute structure: Flack *x* determined using 775 quotients [(*I* ^+^)−(*I* ^−^)]/[(*I* ^+^)+(*I* ^−^)] (Parsons *et al.*, 2013 ▸)
Absolute structure parameter: 0.2 (3)

### 

Data collection: *CrystalClearSM Expert* (Rigaku, 2012 ▸); cell refinement: *CrystalClearSM Expert*; data reduction: *CrystalClearSM Expert*; program(s) used to solve structure: *SUPERFLIP* (Palatinus & Chapuis, 2007 ▸); program(s) used to refine structure: *SHELXL2014* (Sheldrick, 2015 ▸); molecular graphics: *ORTEP-3 for Windows* (Farrugia, 2012 ▸); software used to prepare material for publication: *WinGX* (Farrugia, 2012 ▸).

## Supplementary Material

Crystal structure: contains datablock(s) global, I. DOI: 10.1107/S2056989015011536/tk5369sup1.cif

Structure factors: contains datablock(s) I. DOI: 10.1107/S2056989015011536/tk5369Isup2.hkl

Supporting information file. DOI: 10.1107/S2056989015011536/tk5369Isup3.cml

**Fig. 1.**
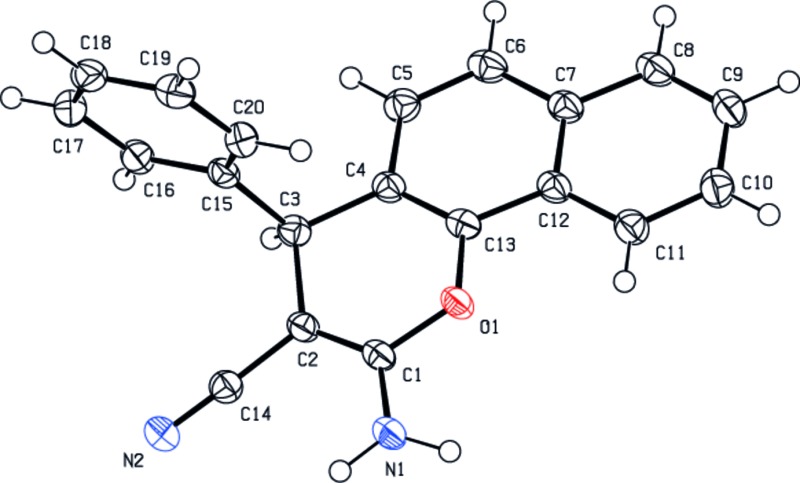
View of the title compound with the atom-numbering scheme. Displacement ellipsoids for non-H atoms are drawn at the 50% probability level.

. DOI: 10.1107/S2056989015011536/tk5369fig1.tif
View of the title compound with the atom-numbering scheme. Displacement ellipsoids for non-H atoms are drawn at the 50% probability level.

**Fig. 2.**
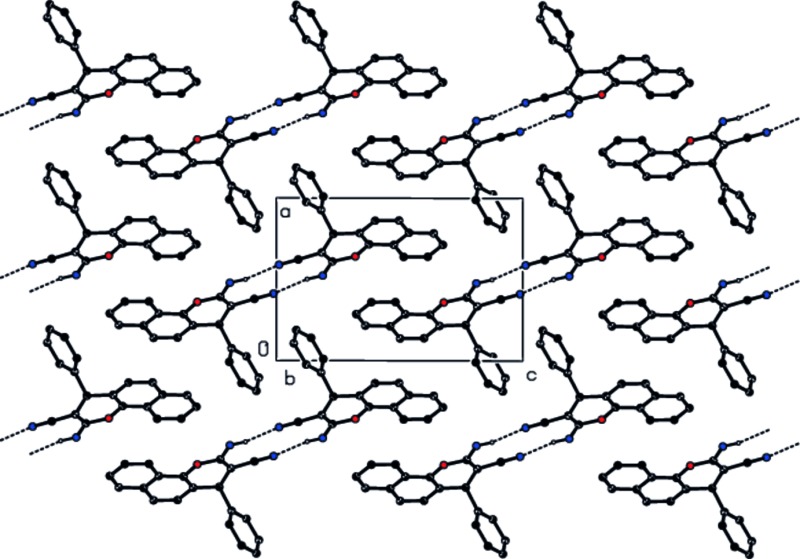
View of the dimers formed by N—H···O hydrogen bonds.

. DOI: 10.1107/S2056989015011536/tk5369fig2.tif
View of the dimers formed by N—H⋯O hydrogen bonds.

CCDC reference: 1406770

Additional supporting information:  crystallographic information; 3D view; checkCIF report

## Figures and Tables

**Table 1:** Hydrogen-bond geometry (, ) *Cg*1 is the centroid of the C15C20 phenyl ring.

*D*H*A*	*D*H	H*A*	*D* *A*	*D*H*A*
N1H1*B*N2^i^	0.91(3)	2.09(3)	2.970(2)	163(3)
C9H9*Cg*1^ii^	0.95	2.88	3.574(2)	131

Symmetry codes: (i) 
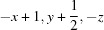
; (ii) 
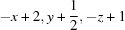
.

## supplementary crystallographic information

### S1. Comment

Among synthetic heterocyclic compounds, aminochromenes represent an important class of organic compounds being the main components of many naturally occurring products (Elagamey *et al.*, 1988; El-Maghraby, 2014). They are used for the chemical synthesis of cosmetics, pigments (Ellis, 1977), and potentially biodegradable agrochemicals (Hafez, *et al.*, 1987). Fused chromene systems have displayed a broad spectrum of biological activities such as mutagenicity (Hiramoto, *et al.*, 1997), sex pheromonal (Bianchi & Tava, 1987), central nervous system (*CNS*) activities (Eiden & Denk, 1991) and inhibitors for influenza virus sialidases (Smith *et al.*, 1998; Taylor *et al.*, 1998). In this context and following our strategy for the synthesis of bio-active molecules, we herein report the synthesis and crystal structure of the title compound.

As seen in Fig. 1, the C4–C13 naphthalene ring system of the title compound is essentially planar [maximum deviations = -0.020 (2) Å for C4 and -0.016 (2) Å for C8]. The C15–C20 phenyl ring makes a dihedral angle of 83.15 (8)° with the mean plane of the naphthalene ring. The 4*H*-pyran ring (O1/C1–C4/C13) in the title compound is puckered [the puckering parameters (Cremer & Pople, 1975) are Q_T_ = 0.177 (2) Å, θ = 98.2 (6) ° and φ = 342.9 (7) °. The structural geometric parameters of the title compound are normal and are consistent with those of the isomer compound 3-amino-1-phenyl-1*H*-benzo[*f*]chromene-2-carbonitrile (Akkurt *et al.*, 2013). Both isomers crystallizes in the same monoclinic space group *P*2_1_ and their unit-cell parameters are almost equal.

In the crystal, pairs of N—H···N hydrogen bonds form inversion dimers with an *R*_2_^2^(12) ring motif (Table 1 and Fig. 2). In addition, C—H···π interactions are observed.

### S2. Experimental

To a solution of 1-naphthol (144 mg; 1 mmol) in 10 ml absolute ethanol, an equimolar amount of benzylidene-malononitrile (154 mg; 1 mmol) was added with constant stirring. The reaction mixture was refluxed for 3 h in the presence of a catalytic amount of piperidine. The reaction progress was monitored by TLC and after cooling, the formed precipitate was filtered off, washed with cold ethanol and dried under vacuum in a desiccator for 24 h. The solid was recrystallized from ethanol. Crystals suitable for X-ray crystallography were obtained by slow evaporation of a solution of the title compound in ethanol (yield 92%; m.p. 483 K).

### S3. Refinement

The H atoms of the NH_2_ group were were refined. The H atoms attached to the C atoms were positioned geometrically, with C—H = 0.95 Å and C—H = 1.00 Å for aromatic and methine H, respectively, and with *U*_iso_(H) = 1.2*U*_eq_(C).

### Crystal data

**Table:**

C_20_H_14_N_2_O	*F*(000) = 312
*M**_r_* = 298.33	*D*_x_ = 1.357 Mg m^−^^3^
Monoclinic, *P*2_1_	Cu *K*α radiation, λ = 1.54178 Å
Hall symbol: P 2yb	Cell parameters from 5573 reflections
*a* = 9.1662 (1) Å	θ = 6.3–66.6°
*b* = 5.7246 (1) Å	µ = 0.67 mm^−^^1^
*c* = 13.9177 (2) Å	*T* = 100 K
β = 90.153 (1)°	Block, brown
*V* = 730.30 (2) Å^3^	0.28 × 0.13 × 0.10 mm
*Z* = 2	

### Data collection

**Table:**

Rigaku AFC11 diffractometer	2201 independent reflections
Radiation source: Rotating Anode	2184 reflections with *I* > 2σ(*I*)
Detector resolution: 22.2222 pixels mm^-1^	*R*_int_ = 0.029
profile data from ω–scans	θ_max_ = 66.7°, θ_min_ = 4.8°
Absorption correction: multi-scan (*CrystalClear-SM Expert*; Rigaku, 2012)	*h* = −10→10
*T*_min_ = 0.883, *T*_max_ = 1.000	*k* = −6→6
5778 measured reflections	*l* = −16→16

### Refinement

**Table:**

Refinement on *F*^2^	Hydrogen site location: mixed
Least-squares matrix: full	H atoms treated by a mixture of independent and constrained refinement
*R*[*F*^2^ > 2σ(*F*^2^)] = 0.031	*w* = 1/[σ^2^(*F*O^2^) + (0.0642*P*)^2^ + 0.1186*P*] where *P* = (*F*_o_^2^ + 2*F*_c_^2^)/3
*wR*(*F*^2^) = 0.093	(Δ/σ)_max_ < 0.001
*S* = 1.09	Δρ_max_ = 0.14 e Å^−^^3^
2201 reflections	Δρ_min_ = −0.15 e Å^−^^3^
216 parameters	Absolute structure: Flack *x* determined using 775 quotients [(*I*^+^)-(*I*^-^)]/[(*I*^+^)+(*I*^-^)] (Parsons *et al.*, 2013)
1 restraint	Absolute structure parameter: 0.2 (3)

### Special details

**Table:**

Geometry. Bond distances, angles *etc*. have been calculated using the rounded fractional coordinates. All su's are estimated from the variances of the (full) variance-covariance matrix. The cell e.s.d.'s are taken into account in the estimation of distances, angles and torsion angles
Refinement. Refinement on *F*^2^ for ALL reflections except those flagged by the user for potential systematic errors. Weighted *R*-factors *wR* and all goodnesses of fit *S* are based on *F*^2^, conventional *R*-factors *R* are based on *F*, with *F* set to zero for negative *F*^2^. The observed criterion of *F*^2^ > σ(*F*^2^) is used only for calculating -*R*-factor-obs *etc*. and is not relevant to the choice of reflections for refinement. *R*-factors based on *F*^2^ are statistically about twice as large as those based on *F*, and *R*-factors based on ALL data will be even larger.

### Fractional atomic coordinates and isotropic or equivalent isotropic displacement parameters (Å^2^)

**Table:**

	*x*	*y*	*z*	*U*_iso_*/*U*_eq_	
O1	0.64120 (15)	0.8755 (3)	0.32053 (9)	0.0250 (4)	
N1	0.5228 (2)	0.9831 (4)	0.18837 (13)	0.0279 (6)	
N2	0.58898 (19)	0.5238 (4)	0.01142 (12)	0.0305 (6)	
C1	0.6097 (2)	0.8195 (4)	0.22720 (13)	0.0223 (5)	
C2	0.6647 (2)	0.6252 (4)	0.18462 (14)	0.0228 (6)	
C3	0.7792 (2)	0.4700 (4)	0.23142 (13)	0.0222 (6)	
C4	0.7851 (2)	0.5235 (4)	0.33777 (13)	0.0225 (6)	
C5	0.8612 (2)	0.3703 (4)	0.40074 (14)	0.0264 (6)	
C6	0.8631 (2)	0.4068 (4)	0.49782 (15)	0.0286 (6)	
C7	0.7877 (2)	0.5997 (4)	0.53815 (14)	0.0255 (6)	
C8	0.7844 (2)	0.6408 (5)	0.63859 (14)	0.0297 (6)	
C9	0.7137 (2)	0.8309 (5)	0.67554 (14)	0.0301 (6)	
C10	0.6403 (2)	0.9868 (5)	0.61433 (15)	0.0307 (6)	
C11	0.6388 (2)	0.9514 (4)	0.51680 (14)	0.0275 (6)	
C12	0.7134 (2)	0.7592 (4)	0.47686 (14)	0.0239 (6)	
C13	0.7165 (2)	0.7126 (4)	0.37642 (13)	0.0226 (6)	
C14	0.6217 (2)	0.5720 (4)	0.08901 (13)	0.0241 (6)	
C15	0.9263 (2)	0.4943 (4)	0.18151 (13)	0.0222 (6)	
C16	0.9754 (2)	0.3182 (4)	0.12136 (13)	0.0261 (6)	
C17	1.1057 (2)	0.3423 (5)	0.07107 (14)	0.0301 (6)	
C18	1.1876 (2)	0.5433 (5)	0.08102 (14)	0.0301 (6)	
C19	1.1396 (2)	0.7208 (5)	0.14112 (14)	0.0293 (6)	
C20	1.0097 (2)	0.6961 (4)	0.19070 (14)	0.0260 (6)	
H1A	0.474 (3)	1.088 (5)	0.2297 (18)	0.034 (7)*	
H1B	0.501 (3)	0.971 (6)	0.125 (2)	0.040 (7)*	
H3	0.74660	0.30430	0.22390	0.0270*	
H5	0.91180	0.24000	0.37480	0.0320*	
H6	0.91520	0.30260	0.53850	0.0340*	
H8	0.83200	0.53470	0.68080	0.0360*	
H9	0.71420	0.85760	0.74290	0.0360*	
H10	0.59130	1.11800	0.64070	0.0370*	
H11	0.58740	1.05650	0.47620	0.0330*	
H16	0.91940	0.17950	0.11430	0.0310*	
H17	1.13830	0.22050	0.03000	0.0360*	
H18	1.27650	0.56020	0.04680	0.0360*	
H19	1.19580	0.85910	0.14820	0.0350*	
H20	0.97710	0.81850	0.23150	0.0310*	

### Atomic displacement parameters (Å^2^)

**Table:**

	*U*^11^	*U*^22^	*U*^33^	*U*^12^	*U*^13^	*U*^23^
O1	0.0306 (8)	0.0258 (8)	0.0186 (6)	0.0031 (7)	−0.0059 (5)	−0.0008 (6)
N1	0.0309 (9)	0.0322 (11)	0.0205 (9)	0.0045 (9)	−0.0056 (7)	−0.0012 (8)
N2	0.0297 (9)	0.0375 (12)	0.0243 (9)	0.0007 (9)	−0.0046 (7)	−0.0041 (8)
C1	0.0219 (9)	0.0273 (11)	0.0178 (8)	−0.0041 (9)	−0.0026 (7)	0.0026 (8)
C2	0.0202 (9)	0.0293 (12)	0.0190 (9)	−0.0025 (9)	−0.0022 (7)	0.0002 (8)
C3	0.0226 (9)	0.0205 (10)	0.0236 (10)	−0.0015 (9)	−0.0021 (7)	−0.0009 (8)
C4	0.0191 (9)	0.0272 (12)	0.0213 (9)	−0.0039 (8)	−0.0007 (7)	0.0010 (8)
C5	0.0249 (9)	0.0270 (12)	0.0272 (10)	0.0003 (10)	0.0001 (7)	0.0025 (9)
C6	0.0276 (10)	0.0325 (13)	0.0256 (10)	−0.0010 (10)	−0.0044 (8)	0.0074 (9)
C7	0.0220 (9)	0.0321 (12)	0.0223 (9)	−0.0050 (9)	−0.0013 (7)	0.0029 (9)
C8	0.0259 (10)	0.0414 (14)	0.0219 (9)	−0.0058 (10)	−0.0031 (8)	0.0058 (9)
C9	0.0275 (10)	0.0427 (14)	0.0200 (9)	−0.0106 (11)	0.0002 (7)	−0.0028 (10)
C10	0.0296 (10)	0.0356 (13)	0.0268 (10)	−0.0044 (11)	0.0024 (8)	−0.0060 (9)
C11	0.0280 (10)	0.0298 (12)	0.0246 (10)	−0.0018 (10)	−0.0018 (8)	−0.0021 (9)
C12	0.0202 (9)	0.0301 (12)	0.0214 (9)	−0.0064 (9)	−0.0012 (7)	0.0006 (8)
C13	0.0214 (9)	0.0251 (11)	0.0213 (9)	−0.0029 (9)	−0.0031 (7)	0.0036 (8)
C14	0.0226 (9)	0.0263 (11)	0.0235 (10)	−0.0010 (9)	0.0001 (7)	0.0003 (9)
C15	0.0227 (10)	0.0265 (11)	0.0173 (8)	0.0004 (9)	−0.0025 (7)	0.0029 (8)
C16	0.0301 (10)	0.0250 (12)	0.0231 (9)	0.0024 (10)	−0.0032 (7)	−0.0016 (8)
C17	0.0319 (11)	0.0346 (13)	0.0238 (9)	0.0069 (10)	0.0008 (7)	−0.0020 (10)
C18	0.0245 (10)	0.0429 (14)	0.0230 (9)	0.0041 (10)	0.0002 (7)	0.0067 (9)
C19	0.0264 (10)	0.0328 (12)	0.0286 (10)	−0.0040 (10)	−0.0030 (8)	0.0056 (9)
C20	0.0274 (10)	0.0263 (12)	0.0244 (10)	0.0001 (10)	−0.0001 (8)	−0.0024 (9)

### Geometric parameters (Å, º)

**Table:**

O1—C1	1.368 (2)	C11—C12	1.410 (3)
O1—C13	1.396 (3)	C12—C13	1.424 (3)
N1—C1	1.342 (3)	C15—C20	1.391 (3)
N2—C14	1.154 (3)	C15—C16	1.386 (3)
C1—C2	1.358 (3)	C16—C17	1.393 (3)
N1—H1B	0.91 (3)	C17—C18	1.381 (4)
N1—H1A	0.95 (3)	C18—C19	1.388 (3)
C2—C14	1.420 (3)	C19—C20	1.385 (3)
C2—C3	1.520 (3)	C3—H3	1.0000
C3—C4	1.512 (3)	C5—H5	0.9500
C3—C15	1.525 (3)	C6—H6	0.9500
C4—C13	1.363 (3)	C8—H8	0.9500
C4—C5	1.422 (3)	C9—H9	0.9500
C5—C6	1.367 (3)	C10—H10	0.9500
C6—C7	1.419 (3)	C11—H11	0.9500
C7—C12	1.422 (3)	C16—H16	0.9500
C7—C8	1.418 (3)	C17—H17	0.9500
C8—C9	1.368 (4)	C18—H18	0.9500
C9—C10	1.404 (3)	C19—H19	0.9500
C10—C11	1.373 (3)	C20—H20	0.9500
			
C1—O1—C13	118.39 (17)	C3—C15—C20	121.36 (18)
O1—C1—N1	110.02 (18)	C16—C15—C20	118.70 (17)
O1—C1—C2	121.91 (18)	C15—C16—C17	120.8 (2)
N1—C1—C2	128.06 (18)	C16—C17—C18	119.9 (2)
C1—N1—H1B	118 (2)	C17—C18—C19	119.82 (18)
H1A—N1—H1B	123 (3)	C18—C19—C20	120.0 (2)
C1—N1—H1A	118.7 (16)	C15—C20—C19	120.8 (2)
C1—C2—C3	123.25 (17)	C2—C3—H3	108.00
C1—C2—C14	118.83 (19)	C4—C3—H3	108.00
C3—C2—C14	117.76 (18)	C15—C3—H3	108.00
C2—C3—C15	111.21 (16)	C4—C5—H5	119.00
C4—C3—C15	113.46 (15)	C6—C5—H5	119.00
C2—C3—C4	108.89 (17)	C5—C6—H6	120.00
C3—C4—C13	122.12 (18)	C7—C6—H6	120.00
C3—C4—C5	119.65 (19)	C7—C8—H8	120.00
C5—C4—C13	118.21 (17)	C9—C8—H8	120.00
C4—C5—C6	121.3 (2)	C8—C9—H9	120.00
C5—C6—C7	120.32 (19)	C10—C9—H9	120.00
C8—C7—C12	118.3 (2)	C9—C10—H10	120.00
C6—C7—C8	122.1 (2)	C11—C10—H10	120.00
C6—C7—C12	119.69 (18)	C10—C11—H11	120.00
C7—C8—C9	120.9 (2)	C12—C11—H11	120.00
C8—C9—C10	120.29 (19)	C15—C16—H16	120.00
C9—C10—C11	120.6 (2)	C17—C16—H16	120.00
C10—C11—C12	120.1 (2)	C16—C17—H17	120.00
C11—C12—C13	122.97 (19)	C18—C17—H17	120.00
C7—C12—C11	119.76 (18)	C17—C18—H18	120.00
C7—C12—C13	117.26 (19)	C19—C18—H18	120.00
O1—C13—C12	114.26 (18)	C18—C19—H19	120.00
O1—C13—C4	122.59 (16)	C20—C19—H19	120.00
C4—C13—C12	123.14 (19)	C15—C20—H20	120.00
N2—C14—C2	178.3 (2)	C19—C20—H20	120.00
C3—C15—C16	119.85 (19)		
			
C13—O1—C1—N1	172.70 (17)	C4—C5—C6—C7	0.3 (3)
C13—O1—C1—C2	−8.3 (3)	C5—C6—C7—C8	178.6 (2)
C1—O1—C13—C4	13.6 (3)	C5—C6—C7—C12	−1.8 (3)
C1—O1—C13—C12	−165.44 (17)	C6—C7—C8—C9	178.5 (2)
O1—C1—C2—C3	−7.7 (3)	C12—C7—C8—C9	−1.1 (3)
O1—C1—C2—C14	176.97 (18)	C6—C7—C12—C11	−179.80 (18)
N1—C1—C2—C3	171.1 (2)	C6—C7—C12—C13	1.3 (3)
N1—C1—C2—C14	−4.2 (3)	C8—C7—C12—C11	−0.2 (3)
C1—C2—C3—C4	16.6 (3)	C8—C7—C12—C13	−179.15 (19)
C1—C2—C3—C15	−109.1 (2)	C7—C8—C9—C10	1.3 (3)
C14—C2—C3—C4	−168.01 (18)	C8—C9—C10—C11	−0.2 (3)
C14—C2—C3—C15	66.3 (2)	C9—C10—C11—C12	−1.1 (3)
C2—C3—C4—C5	167.12 (18)	C10—C11—C12—C7	1.3 (3)
C2—C3—C4—C13	−11.3 (3)	C10—C11—C12—C13	−179.9 (2)
C15—C3—C4—C5	−68.5 (3)	C7—C12—C13—O1	179.84 (17)
C15—C3—C4—C13	113.1 (2)	C7—C12—C13—C4	0.8 (3)
C2—C3—C15—C16	−105.1 (2)	C11—C12—C13—O1	0.9 (3)
C2—C3—C15—C20	71.3 (2)	C11—C12—C13—C4	−178.1 (2)
C4—C3—C15—C16	131.7 (2)	C3—C15—C16—C17	176.54 (18)
C4—C3—C15—C20	−51.9 (3)	C20—C15—C16—C17	0.1 (3)
C3—C4—C5—C6	−176.86 (18)	C3—C15—C20—C19	−176.69 (18)
C13—C4—C5—C6	1.6 (3)	C16—C15—C20—C19	−0.3 (3)
C3—C4—C13—O1	−2.7 (3)	C15—C16—C17—C18	0.1 (3)
C3—C4—C13—C12	176.26 (18)	C16—C17—C18—C19	−0.1 (3)
C5—C4—C13—O1	178.81 (18)	C17—C18—C19—C20	−0.2 (3)
C5—C4—C13—C12	−2.2 (3)	C18—C19—C20—C15	0.3 (3)

### Hydrogen-bond geometry (Å, º)

Cg1 is the centroid of the C15–C20 phenyl ring.

**Table:**

*D*—H···*A*	*D*—H	H···*A*	*D*···*A*	*D*—H···*A*
N1—H1*B*···N2^i^	0.91 (3)	2.09 (3)	2.970 (2)	163 (3)
C9—H9···*Cg*1^ii^	0.95	2.88	3.574 (2)	131

Symmetry codes: (i) −*x*+1, *y*+1/2, −*z*; (ii) −*x*+2, *y*+1/2, −*z*+1.
