# Supplementary figures and images for: Crystal structure of 2-amino-4-phenyl-4H-benzo[h]chromene-3-carbo­nitrile
Source: Acta Crystallogr E Crystallogr Commun. 2015 Jun 27;71(Pt 7):o516–7. doi: 10.1107/S2056989015011536 (PMC4518988; doi:10.1107/S2056989015011536)

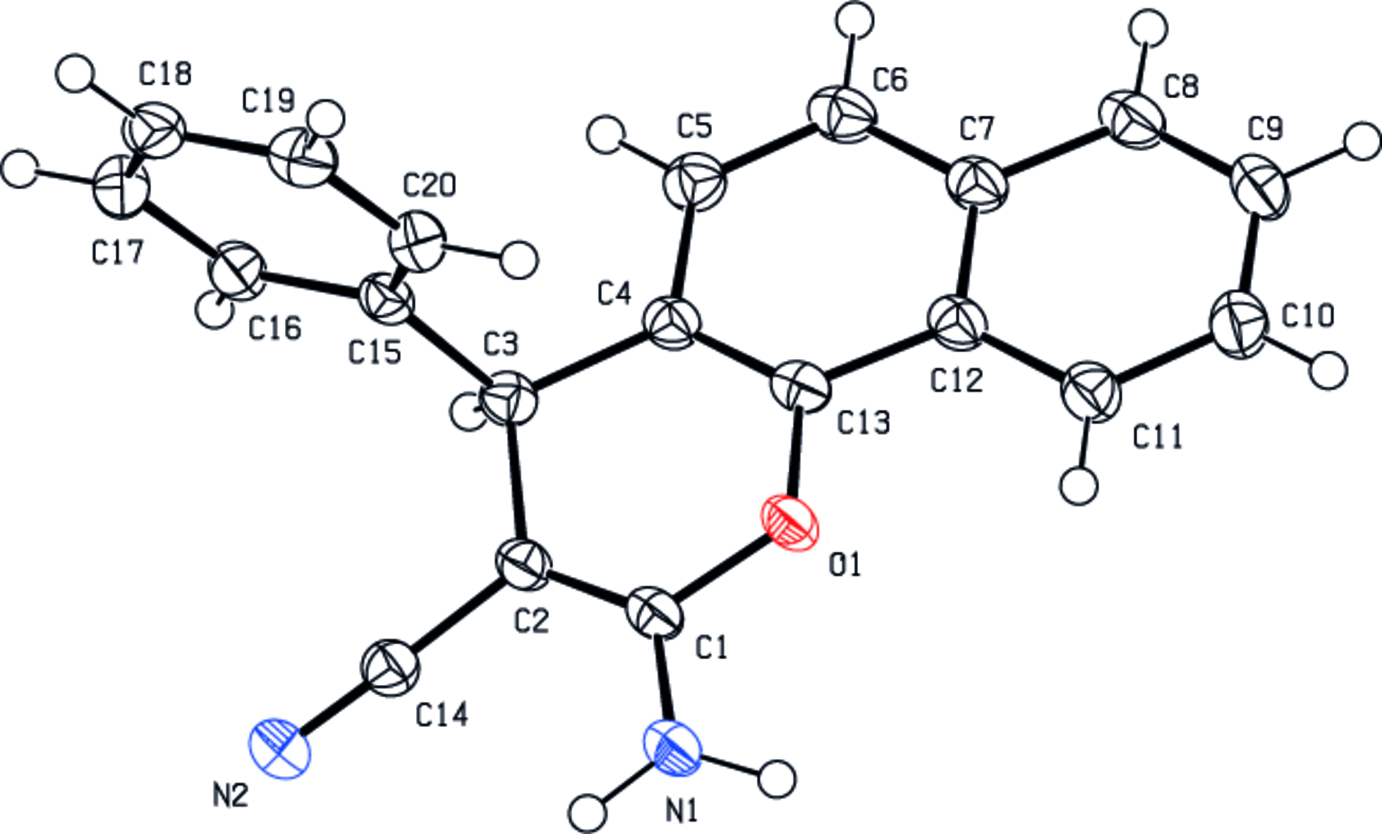

Supplement: Supplementary file 4 [file e-71-0o516-fig1.tif]

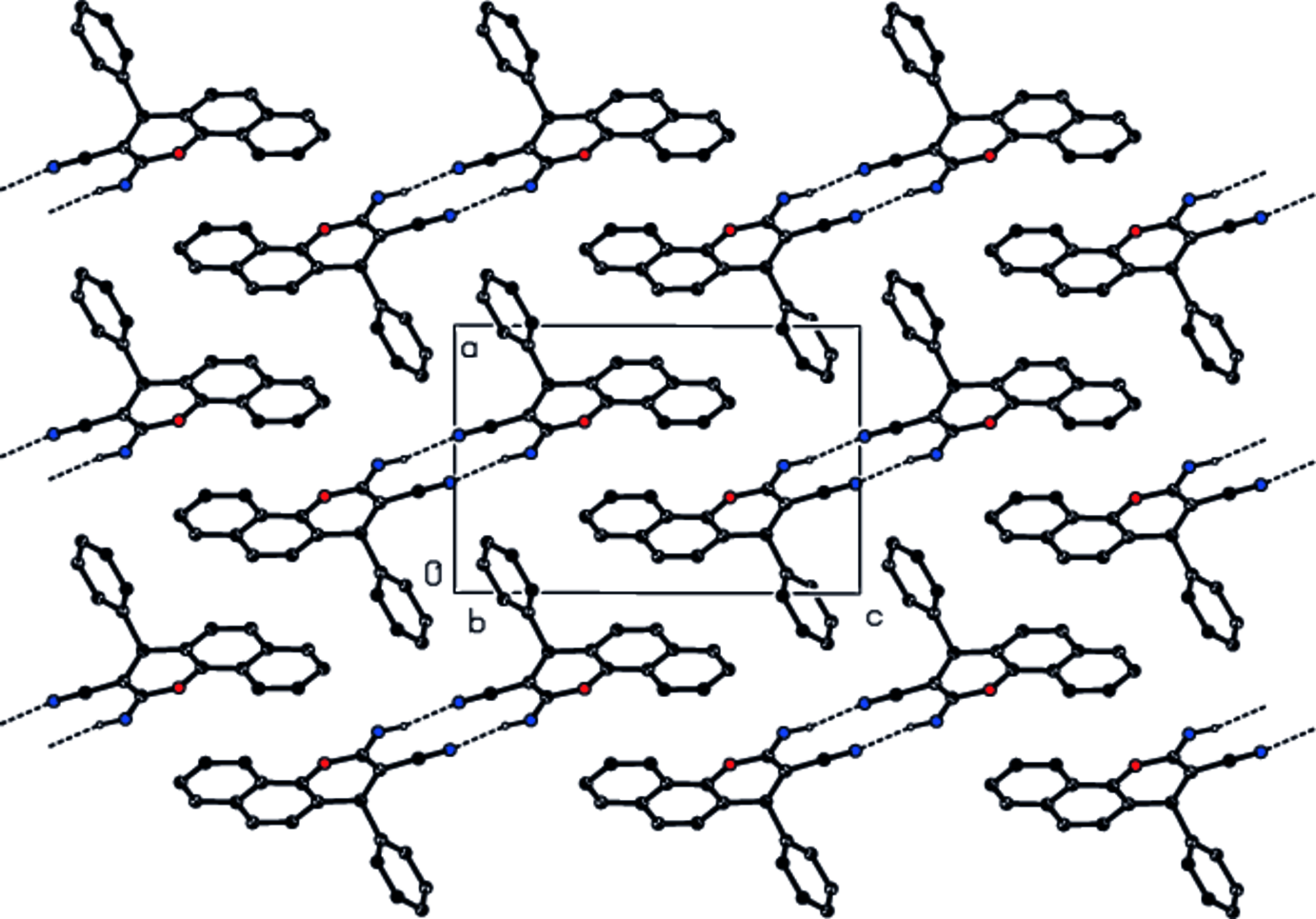

Supplement: Supplementary file 5 [file e-71-0o516-fig2.tif]
